# Unanticipated discovery: incidental encounter with fasciola hepatica during ERCP – a case report

**DOI:** 10.1097/MS9.0000000000002303

**Published:** 2024-06-24

**Authors:** Pasanda Sharma, Prakash Sapkota, Ram Bahadur Gurung, Nikesh Mani Shrestha, Sajan Shrestha, Sankalpa Humagain, Ashish Tamang

**Affiliations:** a Department of Internal Medicine, Gastroenterology and Interventional Endoscopy Unit, Kathmandu University School of Medical Sciences, Dhulikhel Hospital; b Department of Internal Medicine, Kathmandu University School of Medical Sciences, Dhulikhel Hospital, Dhulikhel, Nepal

**Keywords:** anaphylaxis, case report, ERCP, fasciola hepatica, triclabendazole

## Abstract

**Introduction and Importance:**

Fasciola hepatica (FH) is a rare parasitic infection in humans. Its incidental detection during endoscopic retrograde cholangiopancreatography (ERCP) is exceptionally uncommon. This case underscores the importance of considering parasitic infections, even in low-endemicity regions, and the potential implications of dietary and environmental factors in disease transmission.

**Case Presentation:**

The authors present a case of a 31-year-old female from Dhading, Nepal, who underwent ERCP for suspected biliary stone. The patient had been experiencing recurring, nonradiating, burning epigastric pain for 5 to 7 years, which had recently intensified. Previous evaluations, including abdominal ultrasonography, CT, and MRI, revealed a dilation within the common bile duct and an obstruction in the biliary system.

**Clinical Discussion:**

During ERCP, cholangiography revealed mildly dilated extra and intrahepatic bile ducts with irregular filling defects in the common hepatic duct. Sphincterotomy was performed, followed by the extraction of multiple FH worms. A 7 Fr 7 cm double pigtail plastic stent was placed with a good flow of bile. However, the patient experienced anaphylaxis during the procedure, necessitating swift and tailored administration of appropriate medications to ensure effective management and stabilization. The patient was closely monitored in the ICU postprocedure.

**Conclusion:**

After careful monitoring and treatment, the patient fully recovered. The unexpected discovery of FH during ERCP is extremely rare. Early recognition and appropriate management of such incidental findings are crucial to ensuring optimal patient outcomes.

## Introduction

Highlights

- Fasciola hepatica, an uncommon parasitic infection in humans, incidentally encountered during endoscopic retrograde cholangiopancreatography (ERCP), highlighting the rarity of such occurrences.
- The case showcases the diagnostic journey faced with overlapping symptoms, emphasizing the importance of considering parasitic infections, even in low-endemic regions like Nepal. Extraction of the parasite during ERCP led to anaphylaxis, requiring prompt intervention and ICU monitoring, underscoring the challenges in managing unexpected complications.
- Successful management involved biliary stent placement and prescription of Triclabendazole, highlighting the significance of anthelmintic drugs in addressing parasitic infections like Fasciola hepatica.

Fascioliasis continues to be a globally neglected tropical disease with a prevalence of 4.5% in the world, 9% in South America, 4.8% in Africa, and 2% in Asia^1^.

Fasciola hepatica (FH) is a parasitic trematode that primarily affects ruminants but can also infect humans, causing fascioliasis^2^. Humans are only accidental hosts and can harbor adult flukes (measuring 2.5×1 cm) within their biliary tracts, releasing distinctive oval, yellow-brown operculated eggs. These eggs exit the biliary system through stool, undergoing maturation into ciliated miracidia over a span of 2 weeks in freshwater. These miracidia infiltrate intermediate hosts, such as snails from the genus Lymnaea, progressing through the developmental stages of redia and culminating in the cercaria larval stage, featuring a prominent tail for swimming. These cercariae attach to aquatic plant leaves, shed their tails, and encyst into metacercariae, the infective form^2^. Upon ingestion, the cyst wall disintegrates, liberating juvenile flukes into the intestinal lumen. Within hours, they traverse the abdominal cavity, reach the liver within 4–6 days, and embark on a hepatic phase, navigating the liver parenchyma for 6–7 weeks^3^. This phase elicits clinical manifestations, including fever, urticaria, right hypochondriac pain, hepatomegaly, hypergamma globulinemia, and pronounced eosinophilia. Eventually, the flukes transition into their biliary stage, occupying the bile ducts. This stage prompts intermittent right upper quadrant pain, occasionally accompanied by cholangitis or cholestasis. Upon reaching sexual maturity, they lay eggs, perpetuating the cycle^2^. Human infections with FH are relatively rare and are often associated with ingesting contaminated water plants, such as watercress, which act as intermediate hosts for the parasite^2^. The clinical manifestations of fascioliasis in humans vary widely, and the disease can present with vague symptoms or mimic other hepatobiliary disorders, making its diagnosis challenging^4^.

Endoscopic retrograde cholangiopancreatography (ERCP) is a valuable therapeutic tool used for the evaluation and management of various biliary and pancreatic disorders, such as biliary stones, strictures, and tumors^5^. However, the incidental detection of FH during ERCP is exceedingly rare and has been sparsely reported in the medical literature.

Many Nepalese follow traditional agricultural practices and have close associations with livestock^6^. As a result, human infections with zoonotic parasites like FH may be of significant concern in this region. However, there remains a scarcity of literature on fascioliasis in Nepal and other South Asian countries. Understanding the clinical presentation, diagnostic challenges, and management of such cases is vital for enhancing awareness and prompt recognition of this rare parasitic infection.

To the best of our knowledge, there have been only a few documented cases of FH infections in humans undergoing ERCP in Nepal. Therefore, we present this case report involving a 31-year-old female from Nepal who underwent ERCP due to suspicion of biliary stone. During the procedure, an incidental encounter with FH occurred. This case underscores the significance of considering parasitic infections, even in low-endemicity regions. It also emphasizes the necessity for early recognition and appropriate management to ensure optimal patient outcomes.

## Methods

This case report has been structured and presented in accordance with the updated consensus Surgical CAse REport (SCARE, Supplemental Digital Content 1, http://links.lww.com/MS9/A533) guidelines 2023.^7^

## Case description

**Figure 1:**
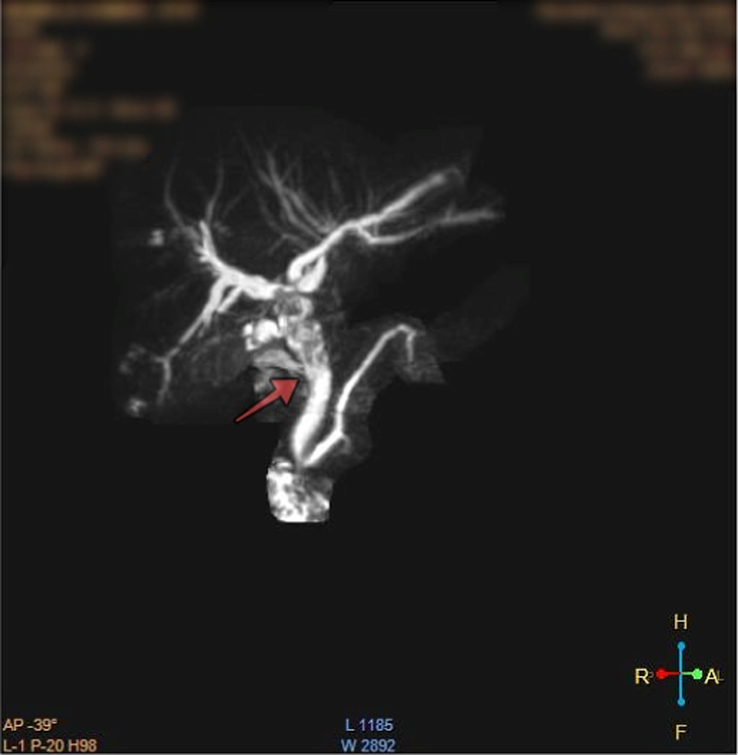
Magnetic resonance cholangiopancreatography (MRCP) image showing mild dilation of extra and intrahepatic bile ducts and irregular filling defects in the common hepatic duct (arrow).

A 31-year-old female hailing from Nilakantha-14, Dhading, a hilly district within the Bagmati province of Nepal was referred to our medical center specifically for an ERCP procedure. The primary reason prompting this referral was her persistent complaints of recurring epigastric pain spanning a duration of 5–7 years, with a recent intensification observed over the past 2–4 days. The nature of her epigastric discomfort was described as nonradiating and of a burning quality. Notably, the pain was exacerbated with fatty and spicy foods and lacked any identifiable alleviating factors. She has a history of drinking water directly from the river. The patient had visited many healthcare facilities for her initial evaluation of her symptoms. During this evaluation, an abdominal ultrasonography revealed a dilation within the common bile duct (CBD). Computed tomography (CT) and MRI, as shown in Figure 1, confirmed the presence of an obstruction in the biliary system as the cause of the symptoms. ERCP procedure was meticulously planned for her case. On physical examination, her vital status was stable, the patient did not appear to be in severe pain. The lungs and heart sounds were normal. The abdomen was flat, and nontender, and bowel sounds were present. Her blood investigation showed raised eosinophils (8.4%); Absolute eosinophil count: 546 cells/μl. However, all the other reports were within normal limits [Table 1].

**Table 1:** Laboratory findings of the patient

Parameters	Value	Normal
Total Leukocyte count (×10^3/μl)	6.5	4.0–11.0
Differential leukocyte count (DLC)		
Neutrophils %	48.6	40–75
Lymphocytes %	40.7	20–45
Eosinophils %	8.4	1–6
Monocytes %	2.3	2–8
Basophils %	00	0–1
Hemoglobin (gm/dl)	12.5	13–17
Platelet count (×103/μl)	469	150–450
PCV	37.1	40–50
Sodium (mmol/l)	141	135–148
Potassium (mmol/l)	4.43	3.5–5.3
Urea (mmol/l)	12.4	10–45
Creatinine (mg/dl)	0.54	0.4–1.1
Random blood glucose (mg/dl)	79.3	60–150
PT (seconds)	12.8	11–13.5
INR (seconds)	1.0	0.9–1.1
Aspartate aminotransferase (U/l)	28.7	5.0–40.0
Alanine aminotransferase (U/l)	24.4	5.0–40.0
Alkaline phosphatase(U/l)	125	<105
Total bilirubin (mg/dl)	0.193	0.3–1.4
Direct bilirubin (mg/dl)	0.097	<0.5

**Figure 2:**
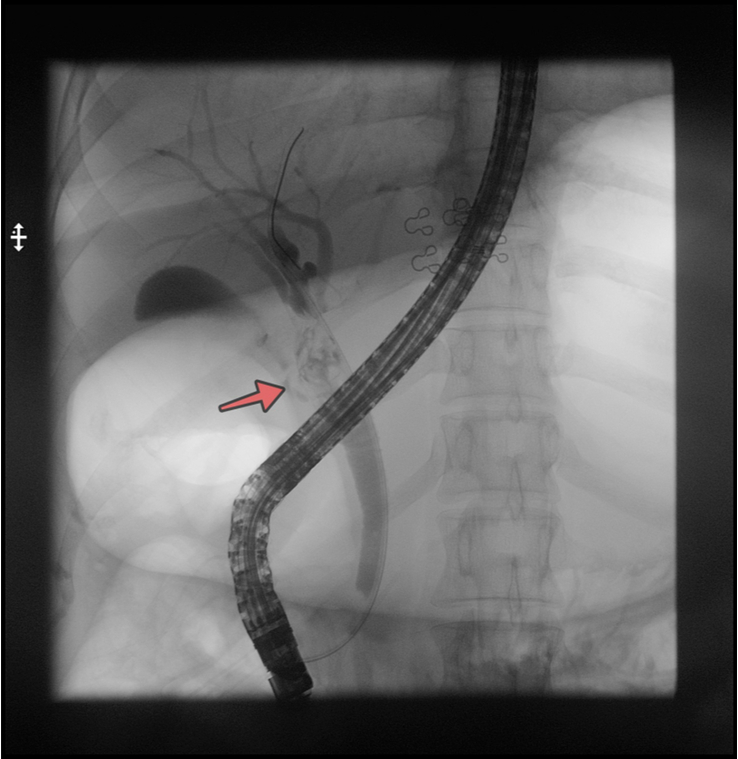
A cholangiogram revealed mildly dilated extra and intrahepatic bile ducts with irregular filling defects in the common hepatic duct (CHD) (arrow).

**Figure 3:**
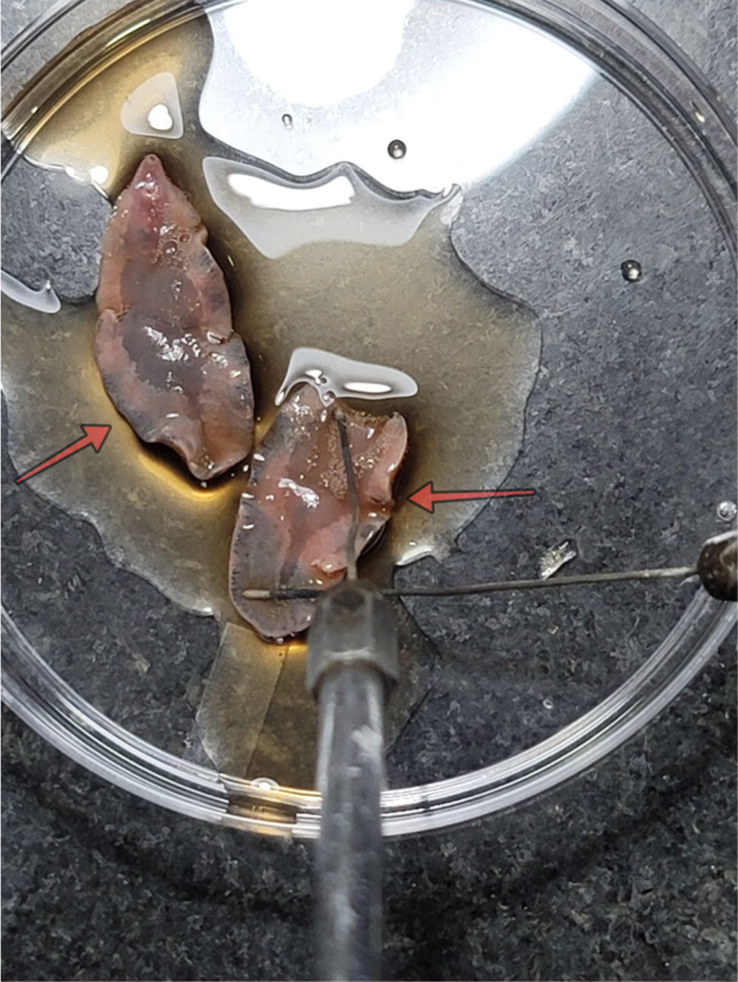
Adult worm Fasciola hepatica measuring 31 mm long by 12 mm wide (arrows).

**Figure 4:**
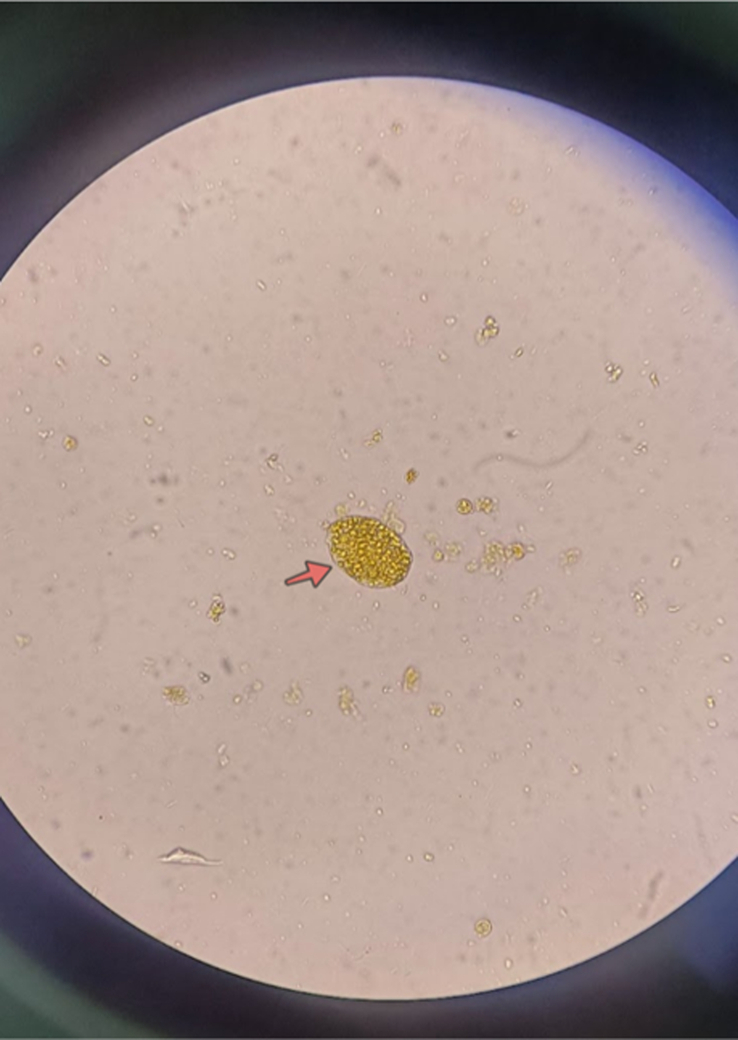
The egg of Fasciola hepatica from the sample collected from the common bile duct (arrows).

ERCP procedure was conducted. The cholangiogram revealed mildly dilated extra and intrahepatic bile ducts with irregular filling defects in the common hepatic duct (CHD), as shown in Figure 2. Sphincterotomy was done followed by basketting, which extracted multiple worms likely FH [Supplemental Digital Content 2, http://links.lww.com/MS9/A534] following which an A 7 Fr 7 cm double pigtail plastic stent was placed with a good flow of bile. The worms were sent to the microbiology laboratory where FH was confirmed both morphologically [Fig. 3 ] and microscopically [Fig. 4]. During the procedure, the patient experienced an episode of anaphylaxis. In response to this critical situation, the medical team promptly administered a combination of medications. These included injections of pheniramine, Adrenaline, hydrocortisone, and phenylephrine, along with intravenous fluids. This swift intervention effectively managed the anaphylactic episode. Given the severity of the anaphylactic reaction, the patient received vigilant monitoring in the ICU after the procedure. With diligent care and a period of observation, the patient’s condition improved. Subsequently, she was discharged on a later day with the expectation of a full recovery. As part of her treatment, she was prescribed Triclabendazole tablets at a dosage of 10 mg/kg, administered in two doses spaced 7 days apart.

## Discussion

Fascioliasis is an important zoonotic infection caused by the liver fluke FH, affecting both humans and animals^2^. In Nepal and other South Asian countries, human fascioliasis has been relatively underexplored, and its prevalence may be underestimated due to the lack of awareness and diagnostic challenges^8^. Human infections often result from the consumption of aquatic plants contaminated with metacercariae, the infective stage of the parasite^2^. Nepal’s agricultural practices and the consumption of raw watercress and other aquatic plants in traditional diets could contribute to the transmission of the parasite in this region. Previous case reports of FH infections in Nepal and other South Asian countries have shed light on the diverse clinical presentations of the disease^9–11^. Similar to our case, patients have presented with nonspecific gastrointestinal symptoms, including abdominal pain, indigestion, and jaundice, often leading to the misdiagnosis of other hepatobiliary disorders. As evident in our case, imaging modalities such as CT and MRI may reveal features suggestive of biliary stones or other obstructive lesions, making the diagnosis of fascioliasis even more challenging^12^. In the realm of clinical practice, discerning between the chronic phase of fascioliasis and other causes of biliary obstruction can be a formidable task due to overlapping symptoms^13^. This diagnostic challenge is amplified in nonendemic areas, where the delayed diagnosis of fascioliasis is not uncommon and symptoms may be mistaken for other hepatic or biliary disorders. Hence, rapid and accurate diagnosis relies on techniques such as ELISA testing, which can provide earlier results compared to direct stool examination^13^.

However, serological tests may not be widely available in resource-limited settings, and their sensitivity and specificity can vary. Additionally, eosinophilia is commonly observed in patients with fascioliasis, as seen in our case, but it is not specific to this infection and can occur in various other parasitic diseases^12^.

**Figure 5:**
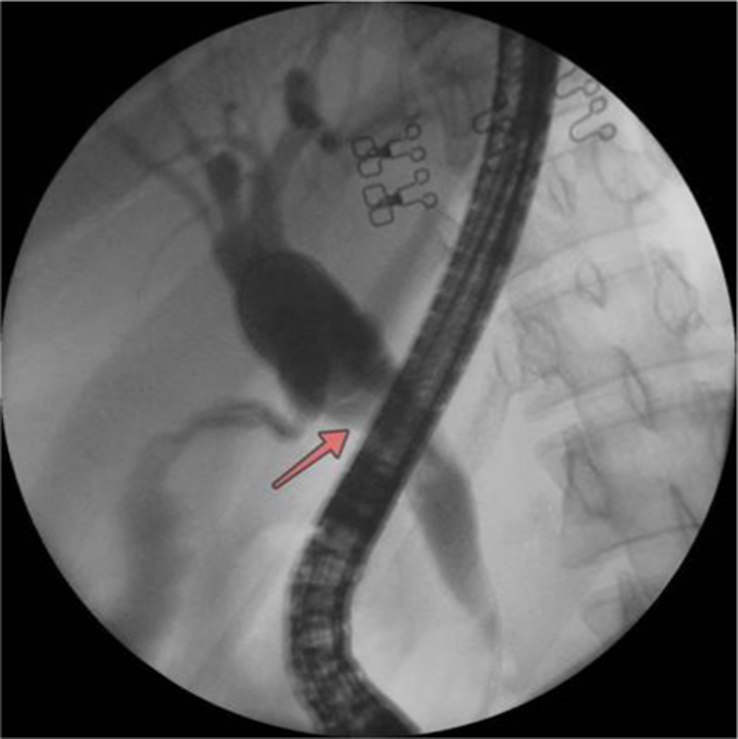
Cholangiogram revealed mildly dilated extra and intrahepatic bile ducts with irregular filling defects in the common hepatic duct (CHD) (arrow).

**Figure 6:**
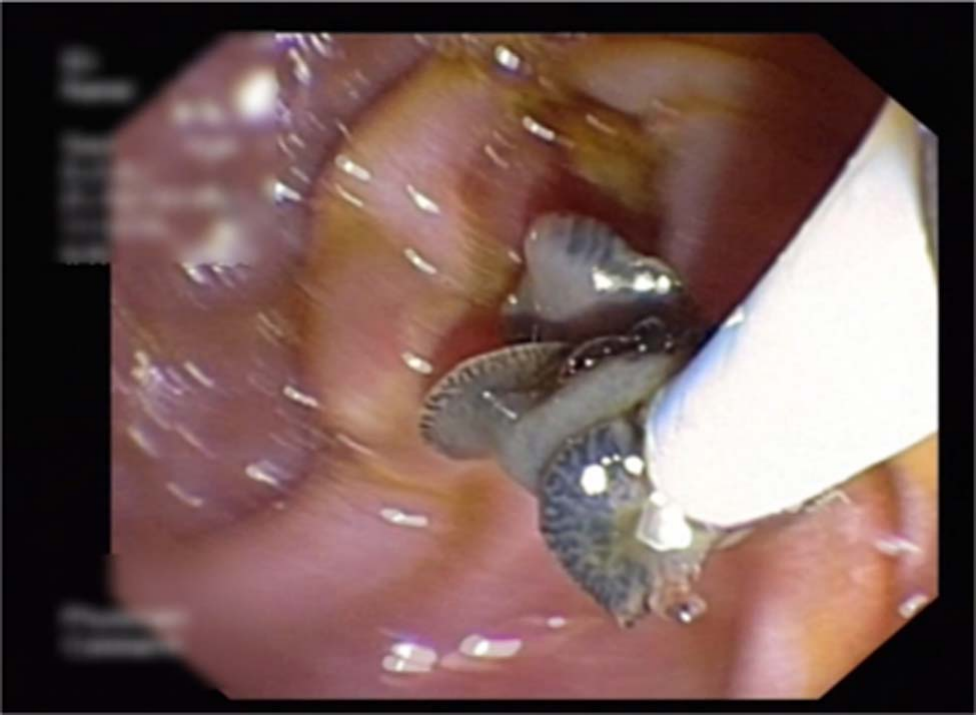
Extraction of Fasciola hepatica.

In a similar event 4 years ago, we encountered a 27-year-old female patient who was referred to our medical center for an ERCP procedure with a suspected choledochal cyst type 1. Prior diagnostic investigations had unveiled an evident dilatation of the proximal segment of the CBD, measuring 16.5 mm, with a transitioning short segment distally, measuring 7.5 mm—a configuration suggestive of choledochal cyst type 1. Notably, her bilirubin levels were elevated, with total bilirubin at 3.1 mg/dl, direct bilirubin at 1.5 mg/dl, ALT at 213 U/l, and ALP at 200 U/l. During the ERCP procedure, a cholangiogram revealed a faint filling defect in the CBD [Fig. 5], leading to the extraction of several liver flukes [Fig. 6], which were subsequently confirmed to be FH. She recovered without any complications during and after the procedure.

Given the rarity of human fascioliasis in Nepal and the broader South Asian region, there is limited data on the optimal management of such cases. Treatment typically involves the administration of anthelmintic drugs such as triclabendazole. However, it is crucial to be cautious during parasite extraction procedures, as seen in our case, as inadvertent parasite rupture may lead to anaphylactic reactions. The use of biliary stents, as performed in this case, can help alleviate biliary obstruction and facilitate the passage of dead or dying parasites.

Treatment strategies, including the administration of triclabendazole 10 mg/kg single dose are recommended in managing Fasciola infection^14^. ERCP may be indicated during the chronic obstructive biliary phase, wherein the extraction of mature FH parasites from the biliary duct becomes a requisite procedure^13,15^. Due to the unavailability of triclabendazole in Nepal, the medication was sourced from a neighboring country. The patient received triclabendazole at a dosage of 10 mg/kg, administered in two doses seven days apart.

Considering the scarcity of reported cases and the potential underestimation of fascioliasis prevalence in Nepal and South Asia, it is essential to raise awareness among healthcare professionals regarding this parasitic infection. Prompt diagnosis, effective management, and patient follow-up are imperative to prevent complications and improve patient outcomes. A collaborative effort involving healthcare providers, parasitologists, and public health authorities is essential to better understand the epidemiology, clinical spectrum, and risk factors associated with human fascioliasis in this region.

## Conclusion

This case report highlights the exceptionally rare occurrence of the incidental detection of FH during ERCP for obstructive jaundice. Early recognition and appropriate management of such incidental findings are essential for optimizing patient outcomes. Clinicians should maintain a high index of suspicion for parasitic infections, even in areas with a low prevalence of such infections. Increased awareness of this potential diagnosis may lead to earlier identification and timely intervention in similar cases. Further studies and case reports are warranted to improve our understanding of this infrequent phenomenon.

## Ethical approval

As the submission is a case report and does not involve any interventional or experimental procedures on patients, ethical approval was not sought. Therefore, no ethical committee approval was necessary for this submission.

## Consent

Written informed consent was obtained from the patient for publication and any accompanying images and videos. A copy of the written consent is available for review by the Editor-in-Chief of this journal on request.

## Source of funding

There were no external sources of funding for this research.

## Author contribution

P.S., P.S., and R.B.G.: patient care, concept, manuscript writing, editing, and review; N.M.S.:. patient care, manuscript writing, editing, and review; S.H.: manuscript writing, data collection, editing, and review; A.T.: patient care, concept, manuscript writing, editing, and review.

## Conflicts of interest disclosure

All authors declare that they have no conflicts of interest in the context of this work.

## Research registration unique identifying number (UIN)

Not applicable as this submission is a case report and does not involve a research study.

## Guarantor

Dr Ram Bahadur Gurung, Head of Department, Internal Medicine Unit Chief, Department of Interventional Gastroenterology, Dhulikhel Hospital.

## Data availability statement

All data generated or analyzed during this study are included in this published article.

## Provenance and peer review

Not commissioned, externally peer-reviewed.

## Patient Perspective

As per the patient’s personal account, she expresses profound gratitude for the timely and effective treatment she received. She conveys her appreciation for finally overcoming the infection and holds the expertise of the medical team in high regard. Her sentiments underline the significance of early intervention, accurate diagnosis, and the crucial role of healthcare professionals in her journey to recovery.

## Supplementary Material
